# Multimodal Imaging in Hereditary Retinal Diseases

**DOI:** 10.1155/2013/634351

**Published:** 2013-04-24

**Authors:** Francesco Pichi, Mariachiara Morara, Chiara Veronese, Paolo Nucci, Antonio P. Ciardella

**Affiliations:** ^1^San Giuseppe Hospital, University Eye Clinic, Via San Vittore 12, 20123 Milan, Italy; ^2^Sant'Orsola-Malpighi Hospital, Ophthalmology Unit, Via Palagi 9, 40138 Bologna, Italy

## Abstract

*Introduction*. In this retrospective study we evaluated the multimodal visualization of retinal genetic diseases to better understand their natural course. *Material and Methods*. We reviewed the charts of 70 consecutive patients with different genetic retinal pathologies who had previously undergone multimodal imaging analyses. Genomic DNA was extracted from peripheral blood and genotyped at the known locus for the different diseases. *Results*. The medical records of 3 families of a 4-generation pedigree affected by North Carolina macular dystrophy were reviewed. A total of 8 patients with Stargardt disease were evaluated for their two main defining clinical characteristics, yellow subretinal flecks and central atrophy. Nine male patients with a previous diagnosis of choroideremia and eleven female carriers were evaluated. Fourteen patients with Best vitelliform macular dystrophy and 6 family members with autosomal recessive bestrophinopathy were included. Seven patients with enhanced s-cone syndrome were ascertained. Lastly, we included 3 unrelated patients with fundus albipunctatus. *Conclusions*. In hereditary retinal diseases, clinical examination is often not sufficient for evaluating the patient's condition. Retinal imaging then becomes important in making the diagnosis, in monitoring the progression of disease, and as a surrogate outcome measure of the efficacy of an intervention.

## 1. Introduction

Evaluation of the retina is a critical step in understanding and diagnosing genetic disease. Because ophthalmologists can view the retina directly, they are often able to make diagnoses without additional testing. In a number of diseases, however, the clinical examination is not sufficient for evaluating the patient's condition. Retinal imaging then becomes important in making the diagnosis and in monitoring the progression of disease. It may be used to document and quantify a patient's symptoms; it may help in making a differential diagnosis of some retinal disorders, in distinguishing localized macular disorders from diffuse retinal disorders, and in distinguishing optic nerve disease from retinal disease. Early alteration of retinal imaging might serve as a surrogate outcome measure of the efficacy of an intervention, rather than waiting for years to determine if a disease has progressed clinically.

Diagnostic imaging procedures used for evaluation and followup of retinal genetic disease include colour fundus photography, blue fundus autofluorescence (FAF) [1–3], fluorescein angiography (FA) [4, 5], indocyanine green angiography (ICGA) [6], and spectral-domain optical coherence tomography (SD-OCT) [5, 7, 8]. 

We evaluate here the multimodal visualization of retinal genetic diseases and correlate FAF, FA, and ICG of study lesions with SD-OCT to assess their ultrastructural characteristics and to better understand the natural course of the diseases [7].

## 2. Methods

We reviewed the charts of 70 consecutive patients with different genetic retinal pathologies visited at the Ocular Genetic Department of the Sant'Orsola-Malpighi Hospital, Bologna, between January 2008 and December 2012. Only eyes having previously undergone multimodal imaging analyses (high-resolution digital colour fundus photographs, FAF, FA, ICGA, and SD-OCT) were included. All patients underwent a complete ophthalmologic examination, including measurement of best-corrected visual acuity (BCVA) using standard Early Treatment of Diabetic Retinopathy Study (ETDRS) charts, slit lamp examination of the anterior segment, and fundus biomicroscopy. Colour fundus photographs were obtained using a high-resolution digital retinal camera (Topcon TRX-50 retinal camera; Tokyo, Japan). For high-resolution multimodal fundus imaging analysis, we used a combined instrument (Spectralis HRA-OCT, Heidelberg Engineering, Heidelberg, Germany) that allows for simultaneous recording of cSLO and SD-OCT images. A standardized imaging protocol was performed in all patients, which included acquisition of blue FAF and FA (excitation *k* = 488 nm; emission *k* > 500 nm; field of view, 30° · 30°; image resolution 768 · 768 pixels), simultaneous SD-OCT scanning using a second, independent pair of scanning mirrors (*k* = 870 nm; acquisition speed, 40.000 A-scans per seconds; scan depth, 1.8 mm; digital depth resolution, approximately 3.5 *μ*m per pixel; optical depth resolution, 7 *μ*m; lateral optical resolution, 14 *μ*m), and indocyanine green angiography (excitation *k* = 787 nm; emission *k* > 800 nm; field of view, 30° · 30°; image resolution, 768 · 768 pixels) [7]. Fundus autofluorescence, FA, ICGA, and SD-OCT images were evaluated on a computer monitor by two independent examiners (F.P. and M.M.). 

Genomic DNA was extracted from peripheral blood with the Qiagen Blood DNA extraction kit (Qiagen, Crawley, UK) and genotyped at the known locus for the different diseases using short tandem repeat polymorphisms [9]. DNA samples were subsequently examined with a genome-wide scan of single nucleotide polymorphisms and the genotypes that were produced were studied with linkage and haplotype analyses [10].

## 3. Results

### 3.1. North Carolina Macular Dystrophy (NCMD)

**Figure 1:**
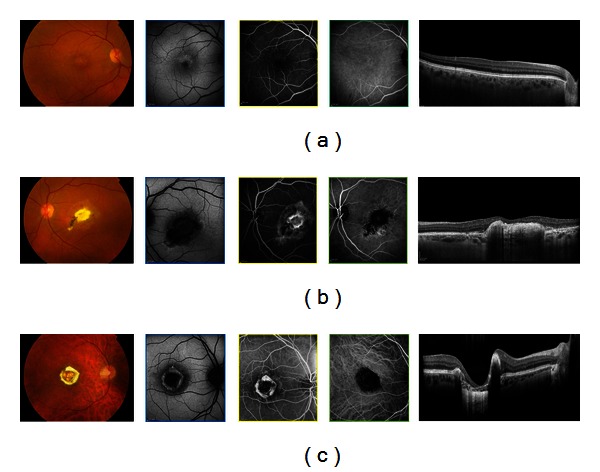
Fundus photographs of affected patients with North Carolina macular dystrophy, showing fine, confluent drusen in the macula (grade I), a subretinal scar due to a prior choroidal neovascularization (Grade II), and a macular caldera (Grade III). In Grade I lesion, autofluorescence shows marked hyperautofluorescence of the bright drusen-like elements. SD-OCT shows a normal anatomy of the inner retina; the photoreceptor-retinal pigment epithelium complex appears normal, and the drusen-like lesions are not detectable. In Grade II lesion, SD-OCT shows increased reflectivity consistent with subretinal fibrosis or gliosis. In the Grade III lesion, fundus autofluorescence demonstrates central hypoautofluorescence, surrounded by normal appearing retina tissue and preserved foveal tissue. There is a hyperautofluorescent perimeter surrounding the edges of the lesion, possibly indicative of metabolic byproduct deposition. SD-OCT demonstrates a normal hyperreflective IS/OS photoreceptor junction with abrupt attenuation of outer retinal structures and RPE absence within the lesion.

The medical records of 3 families of a 4-generation pedigree were reviewed; the first recorded examination period took place in 2009, and the last took place in 2012. Twelve members were found to be clinically affected with NCMD. Macular findings among the affected patients ranged from disease grade 1 (6 eyes, mean age 57 years old) to grades 2 (8 eyes, mean age 44 years old) and 3 (10 eyes, mean age 19 years old) (Figure 1). All affected patients had symmetric disease grades bilaterally; when first examined, those with disease grade 2 had a median VA of 20/25 and those with disease grade 3 had a median VA of 20/60. In addition, all affected family members had numerous yellow, midperipheral drusen-like deposits in both eyes. 

Genetic testing of family members mapped the responsible mutation to the MCDR1 locus on chromosome 6 between the markers D6S1609 and D6S1627. 

Grade 1 lesions showed clusters of peculiar, yellow-white spots at the level of the RPE; angiography revealed a pattern of hyperfluorescence that was apparent in the arterial phase and did not change during the course of angiography. The drusen-like lesions could not be picked up by SD-OCT, but where hyperautofluorescent, suggesting a lipofuscin composition (Figure 1(a)).

Grade 2 lesions consisted of substantial disciform scarring, often resulting from CNV; autofluorescence imaging showed a ring of increased autofluorescence, surrounded by a ring of decreased autofluorescence corresponding to the ring of mottled RPE. Spectral domain OCT showed loss of architecture of the outer retina due to fibrotic CNV, without activity on FA (Figure 1(b)). 

Grade 3 lesions consisted of excavated atrophic chorioretinal lesions with an overhanging lip of retina that partly surrounded these lesions; these features were confirmed by autofluorescence, that showed loss of central autofluorescence with a ring of hyperautofluorescence at the edge of the lesions. Angiography revealed the presence of only a few small choroidal blood vessels in the area of the macular lesions, surrounded by subretinal fibrosis (Figure 1(c)).

### 3.2. Stargardt Disease

A total of 16 eyes (8 patients) with Stardardt disease were retrospectively reviewed; there were 5 women and 3 men with a mean age at presentation of 39 years (range 17–46 years). Best-corrected visual acuity ranged from 20/20 to 20/400, with a mean of 20/80.

Two main defining clinical characteristics of the disease were identified in our patients: 7 eyes (43.7%) presented with yellow subretinal flecks, 5 eyes (31.2%) showed central atrophy, and 4 eyes (25.1%) a combination of the previous lesions. 

**Figure 2:**
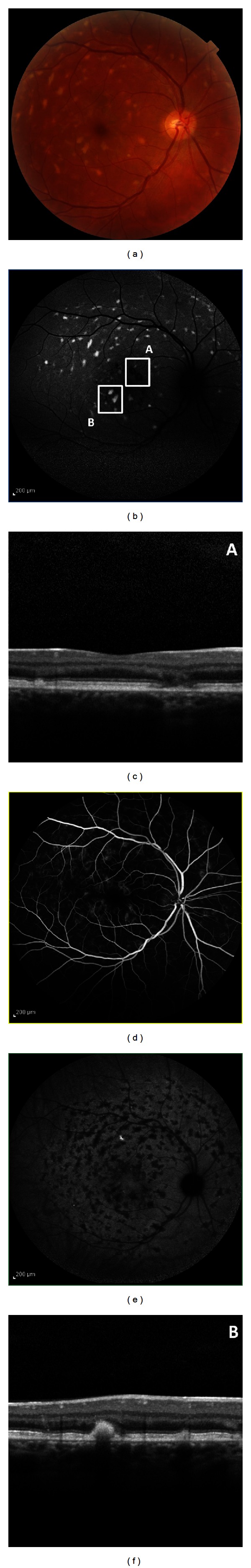
Color photograph shows macular atrophy and retinal flecks in a Stargardt patient. OCT scans are illustrated by the arrows 1 and 2. The autofluorescent frame clearly delineates the retinal flecks. Thin and open arrows point to two of the flecks, respectively, crossed by scans 1 and 2. Fluorescein angiography shows the dark choroid appearance, and the hypofluorescent flecks are hardly discernable in the hyperfluorescent background because of RPE changes. On ICG, late phase (30 min), the hypofluorescent lesions appear more numerous than the autofluorescent flecks, and there is sparing of the peripapillary area. The SD-OCT scan in square A shows a small hyperreflective lesion located at the inner part of the RPE layer, called type 1 deposit, and macular atrophy. The SD-OCT scan in square B shows a small hyperreflective linear lesion located at the level of the outer nuclear layer and clearly distinguished from the RPE layer, called type 2 deposit.

On colour fundus photography, the retinal flecks presented heterogeneous patterns, which were perifoveal or widely distributed in the fundus (Figure 2). Flecks corresponded on FAF imaging to areas where the autofluorescence signal differed from the overall background level (Figure 2(b)). Most flecks seen on FAF were hyperautofluorescence, whereas some were surrounded by a ring of decreased autofluorescence. On FA the characteristic pisiform flecks were hypo- or hyperfluorescent (Figure 2(d)). Spectral Domain OCT scans through the flecks clearly revealed 2 patterns, as described by Querques et al. [11]. Type 1 deposits are dome shaped and located at the level of or just above the RPE; type 1 deposits were observed in 91% of our patients (Figure 2(b), square a). Type 2 deposits presented as small, linear hypoerreflective lesions located at the level of the inner segments of photoreceptors or outer nuclear layer (ONL) and clearly separated from the RPE; type 2 lesions were observed in 84% eyes (Figure 2(b), square b). The number of flecks located in the macula increased radially exhibiting a pattern of centrifugal addition beginning from the fovea and extending toward the outer edges of the macula; newer flecks appeared hyperautofluorescent on FAF imaging, while older flecks become progressively more hypoautofluorscent with time. 

Central atrophy corresponded to a clearly demarcated area of uniform decreased autofluorescence surrounded by a border of patchy, mottled hypoautofluorescence (Figure 2(b)); the sharply demarcated areas of central atrophy gradually enlarged in 3 eyes, and new areas of atrophy appeared to emerge and extend selectively into locations demonstrating mottled hypoautofluorescence. Angiography demonstrated perifoveal window defect, corresponding to dropout of RPE cells, and visualization of the large choroidal vessels centrally (Figure 2(d)). 

An angiographically dark choroid was noted in 62% of our patients (Figure 2(d)); the lack of early choroidal flush is thought to be due to blocking of choroidal fluorescence by a diffuse accumulation of lipofuscin in the RPE.

### 3.3. Choroideremia

**Figure 3:**
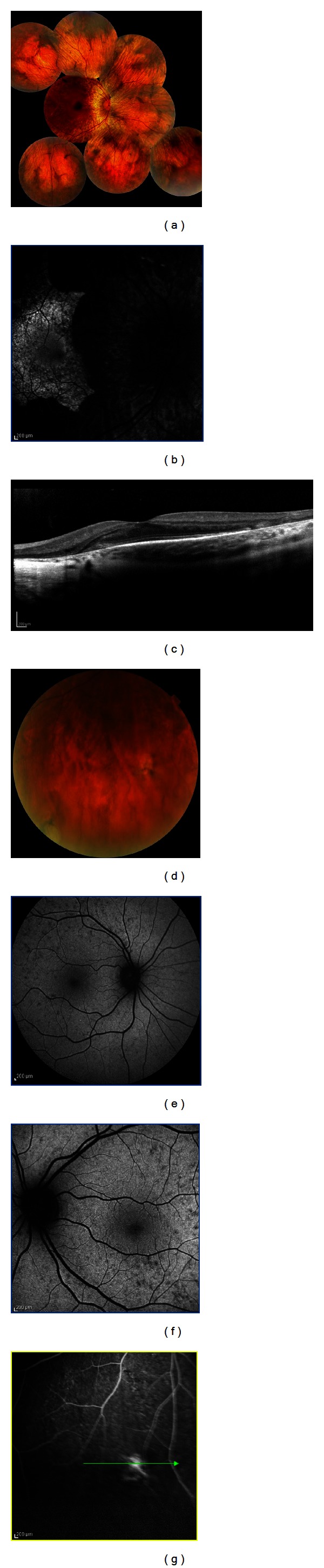
Fundus photograph of a male patient affected by choroideremia shows midperipheral RPE atrophy spreading peripherally with visualization of coursing large choroidal vessels and sparing of the fovea. Fundus autofluorescence demonstrates an hyperautofluorescent stellate preservation of the posterior pole. Spectral-domain OCT scans show diffuse choroidal atrophy and loss of the IS/OS junction, which is intact and preserved at the level of the fovea. The boy's mother was a genetically confirmed female carrier: her fundus shows mild, patchy peripheral RPE mottling with a striking speckled pattern in FAF.

Medical records of nine male patients with a previous diagnosis of choroideremia were reviewed; mean age was 23 years old, and mean BCVA at presentation was 20/30. The disease was bilateral and symmetric in 9/9 (100%) patients. Three patients (33.3%) showed midperipheral RPE abnormalities, which in 5 patients (55.5%) had already spread peripherally with involvement of the choroid too and visualization of coursing large choroidal vessels; one patient (11.1%) presented with vascular attenuation and optic atrophy (Figure 3). The fovea was spared in every case. In 100% of the patients, FA showed filling of the retinal and large choroidal vessels, but not of the choriocapillaris. The intact fovea was hypofluorescent and surrounded by a hyperfluorescence stellate preservation of the posterior pole due to an extensive window defect. Zonal areas of peripheral atrophy were accentuated on the FAF as areas of hypoautofluorescence, which contrasted with the physiological autofluorescence of the fundus (Figure 3(b)). Spectral domain OCT scans showed diffuse choroidal atrophy and loss of the IS/OS junction, which was intact and preserved at the level of the fovea (Figure 3(c)).

Eleven female carriers of the 9 independent families were examined, 9 mothers and 2 sisters. Mean BCVA was 20/25. All carriers (100%) showed mild, patchy peripheral RPE atrophy and mottling with a striking speckled pattern in FAF and simultaneous choroidal and retinal filling in FA (Figures 3(d), 3(e), 3(f), and 3(g)).

### 3.4. *Bestrophinopathies*

Fourteen patients with Best vitelliform macular dystrophy (BVMD) were included. The mean age of enrolled subjects was 37 years (range 9 to 59 years). The median BCVA was 20/50 (range 20/20 to 20/320). 

**Figure 4:**
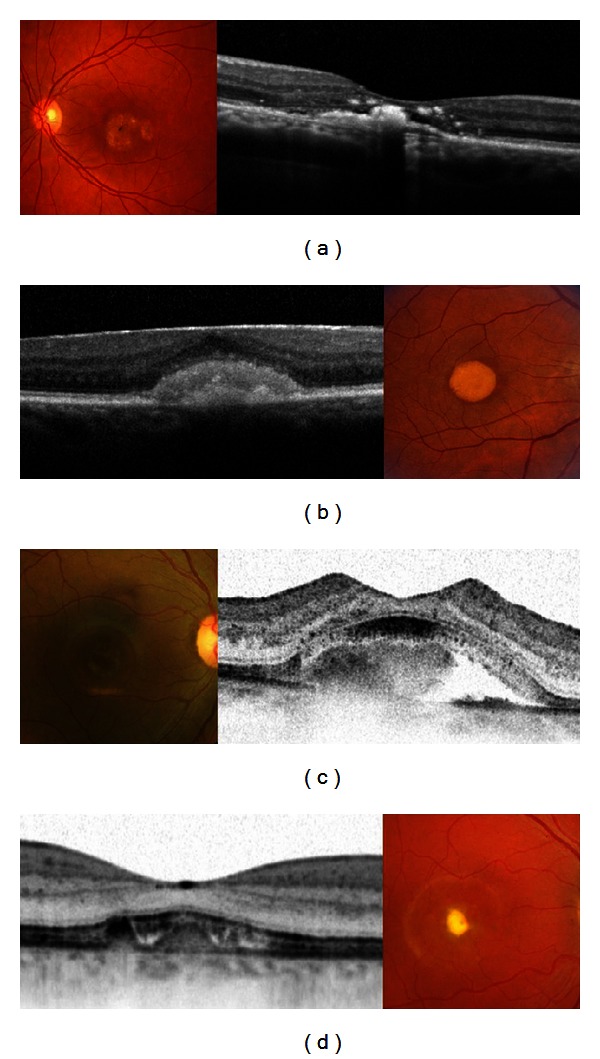
Spectral domain-OCT scans document the natural evolution of BVMD. Firstly a thicker layer between the photoreceptors inner/outer segment interface and the underlying RPE (previtelliform stage). The material is subsequently deposited under the neurosensory retina (vitelliform stage) and is sometimes accompanied with a neurosensory detachment (pseudohypopyon stage) secondary to its reabsorption, with abnormalities of the RPE and subsequent thinning of all retinal layers (vitelliruptive and atrophic stage).

In 2 patients with a positive family history of BVMD, clinical evaluation showed RPE mottling and SD-OCT imaging demonstrated a prominent highly reflective material between the RPE/Bruch's membrane complex and the IS/OS junction (previtelliform stage, Figure 4(b)).

In 5 patients, an elevated yellow round macular lesion measuring approximately 3/4 disc diameter across was found in the fundus. On SD-OCT imaging, the foveal retina was elevated due to a homogeneous hyperreflective material located just above the RPE. The hyperreflective material presented increased fluorescence on FAF (vitelliform stage, Figure 4(a)). 

In 4 patients, an elevated oval macular lesion measuring approximately three disc diameters across was observed in both eyes. A yellowish material located more prominently at the inferior aspect of the lesion was evident. In the center of the macula, SD-OCT imaging showed separation of the neurosensory retina from the RPE by an optically clear space and clumps of homogeneous hyperreflective material overlying the RPE inferiorly displaced. On FAF, increased autofluorescence from the hyperreflective material overlying the RPE was observed from the inferior aspect of the lesion (pseudohypopyon stage, Figure 4(c)).

In 3 patients elevated macular lesions, with variable amounts of yellowish subretinal material dispersed within, were observed. SD-OCT imaging showed the neurosensory retina separated from the RPE centrally by an optically clear space, photoreceptors' OS irregularly elongated, and hyperreflective mounds at the level of the RPE, clearly delineated on FAF (vitelliruptive stage, Figure 4(d)).

**Figure 5:**
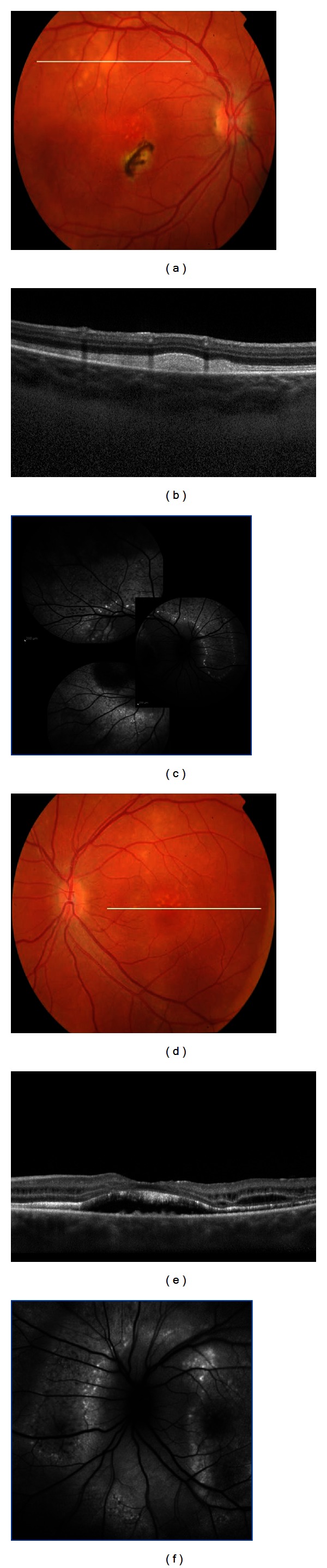
Fundus photograph of an autosomal recessive bestrophinopathy shows a well-demarcated area with round yellowish-white deposits at the posterior pole and along the superotemporal arcade; subretinal fibrosis is visible inferior nasal of the fovea. SD-OCT scan illustrates extensive RPE deposits extending to the outer plexiform layer. Fundus autofluorescence imaging displayed marked hyperautofluorescence corresponding to the yellow lesions. Fundus photograph shows a well-demarcated area with round yellowish-white deposits at the posterior pole extending to the superior periphery. SD-OCT scan demonstrates RPE detachment from the photoreceptors. Photoreceptor outer segments are thickened and elongated. Fundus autofluorescence identified additional peripapillary hyperfluorescent deposits not seen on funduscopy.

Two of the previous patients had family members with DNA samples available for study, and in each of these families, the disease causing variations were found to lie on separate alleles, consistent with autosomal recessive bestrophinopathy (ARB). Six patients with ARB from 2 nonconsanguineous families were therefore reviewed. All patients had bilateral maculopathy with subretinal yellow deposits. Subretinal deposits in subjects 3 to 6 were more prominent, appearing as multifocal, round, yellow lesions surrounding a macular neurosensory retinal detachment (Figures 5(a) and 5(d)). Similar additional areas of 2 to 3 disc diameters were present above the optic discs. In all patients, fundus autofluorescence imaging displayed marked hyperautofluorescence corresponding to the yellow lesions (Figures 5(c) and 5(f)). In subject 2, this identified additional peripapillary hyperfluorescent deposits not seen on funduscopy. Spectral-domain optical coherence tomography scans demonstrated round- or dome-shaped deposits within the RPE reaching into the subretinal space in all subjects, which extended up to the OPL (Figure 5(b)). The RPE itself was thinned throughout the macula. Photoreceptor outer segments were thicker and elongated compared with normal control data. Small filament-like bridges were visible between the photoreceptor outer segment layer and the RPE (Figure 5(e)). Verhoeff's membrane, the interface between cone photoreceptors and the RPE, was not identifiable in multiple scans. Inner retinal layers were unaffected. All subjects harbored compound heterozygous mutations in BEST1; the 2 most frequent recessive alleles observed in our series were Arg141His (3 patients) and Ala195Val (2 patients), whereas one patient had an Arg92Cys mutation.

### 3.5. Enhanced S-Cone Syndrome (ESCS)

**Figure 6:**
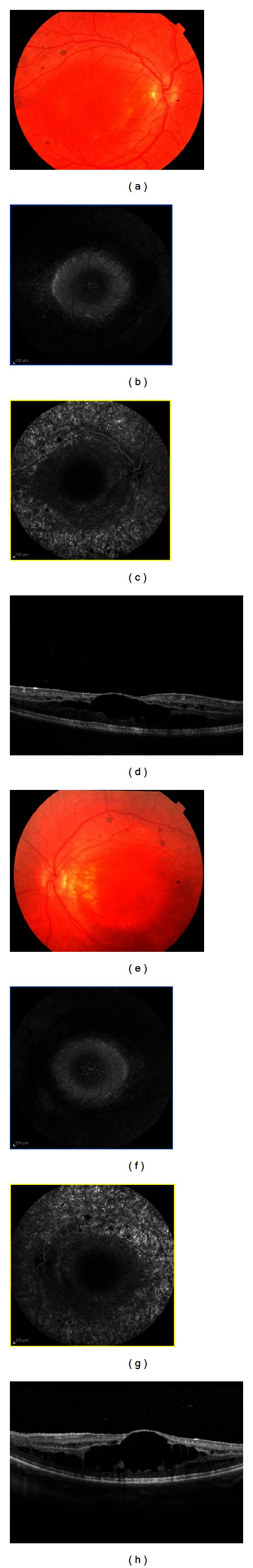
Color fundus photography of a patient with enhanced s-cone syndrome showing nummular pigmentary clumping at the level of the RPE along the vascular arcades and macular disturbance with subtle pigmentary changes; fundus autofluorescence shows an absence of AF outside the arcades and a ring of increased AF at the posterior pole. Foveal schisis is demonstrated on SD-OCT and confirmed by the absence of leakage on fluorescein angiography.

Seven patients with ESCS were ascertained. The clinical presentation in all patients was night blindness, with or without reduced central vision. Nyctalopia had begun in early childhood with onset ranging from 2 to 6 years of age. No patient reported photophobia. Visual acuity ranged between 20/20 and 20/80. Nystagmus was not present in any patient. Vitreous cells were present in all patients. The fundus appearance varied between patients. The youngest subject had normal appearing fundi. Six patients had subtle to mild pigmentary changes with nummular pigmentary deposition at the level of the RPE, usually located in the midperiphery along the vascular arcades and often associated with RPE atrophy, with one of these subjects having small yellow-white dots at the posterior pole (Figures 6(a) and 6(e)). Foveal schisis-like changes were observed in 6 patients. OCT demonstrated foveal cyst formation (Figures 6(d) and 6(h)). Subsequently, these patients had FA, which showed no leakage, suggesting that the cystoid changes were more likely to be secondary to schisis than to edema (Figures 6(b) and 6(f)). Fundus autofluorescence showed a decrease or lack of autofluorescence outside the arcades, a ring of relatively increased autofluorescence in the transitional zone between this area of decreased/absent autofluorescence and the macular region, and a spoke-like area of relatively increased autofluorescence centered on the fovea (Figures 6(c) and 6(g)). Mutations were identified in 7 of 7 subjects. Three previously reported mutations were identified, a splice acceptor intron 1 mutation (IVS1-2A>C splice site) with the Arg311Gln (R311Q) and Arg104Trp (932G→A; exon 6substitutions) and the 481delA frameshift mutation.

### 3.6. Fundus Albipunctatus

We included 3 unrelated patients (aged 20, 35, and 8 years) with fundus albipunctatus.

**Figure 7:**
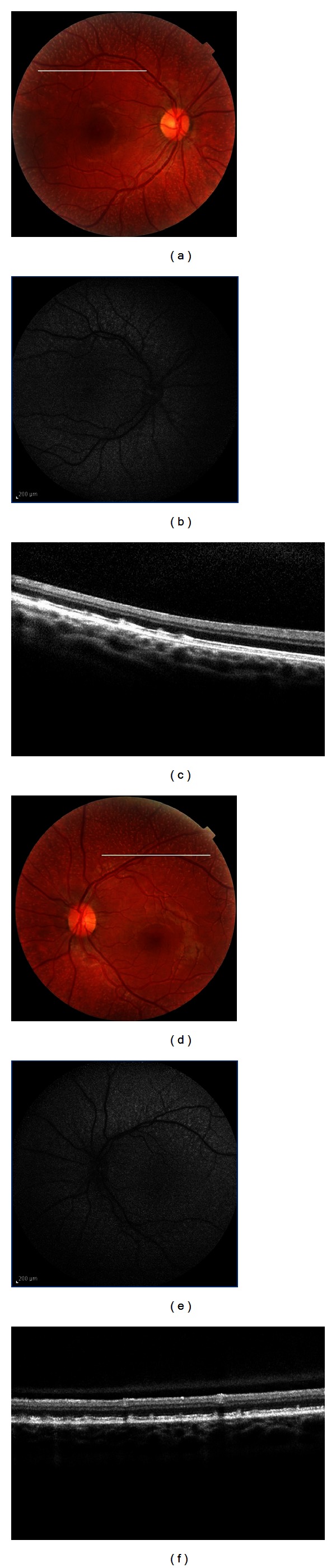
Fundus photograph of two eyes with fundus albipunctatus shows many small white dots scattered throughout the retina except for the foveal area. Fundus autofluorescence imaging of both eyes produced fuzzy and grainy images, and the margins of the optic nerve head and blood vessels are blurred. On SD-OCT, focal thickening centered in the photoreceptor OS and extending forward close to the outer limiting membrane and backward to the outer aspect of the RPE is seen corresponding to the multiple discrete albipunctate dots. These lesions seem to bulge their outer and inner boundaries. There was a reduced visualization of the photoreceptor outer tips.

All patients had diffusely scattered white round flecks scattered in a radial pattern from the vascular arcades throughout the retina, except for the foveal area (Figures 7(a) and 7(d)). Mean BCVA was 20/25, and the results of the ocular examination were normal except for the fundus. Spectral domain OCT revealed an irregular IS band through the macula and a dome-shaped elevation corresponding to discrete albipunctate dots (Figure 7(c) and 7(f)). These elevated lesions were at the level of the RPE and extended into the IS band and external limiting membrane. The ONL thickness was thinner than that in normal controls. In 2/3 patients the FAF image was fuzzy and grainy, and the margins of the optic nerve head and blood vessels were blurred. No enhanced hyperautofluorescent spots or rings, which may correlate to the albipunctate spots on the fundus photo, were observed. Whereas fundus autofluorescence was scarcely detectable in the 2 oldest patients, it was severely reduced in the youngest (Figures 7(b) and 7(e)) but still detectable. 

Two patients harbored homozygous mutations (c.881G.C and c.382G.A, resp.) and the third patient harbored the compound heterozygous mutations (c.95delT and c.712G.T) in RDH5.

## 4. Discussion

This study reports a comparison between FAF, FA, ICGA, and OCT for imaging of genetic retinal diseases.

North Carolina macular dystrophy is an autosomal dominant macular dystrophy with variable expressivity but complete penetrance that rarely progresses [12, 13]. Visual acuity is often better than expected from the clinical examination of the macula, ranging from 20/20 to 20/200, with a median of 20/50. Color vision, full-field electroretinography, and electrooculography results typically are normal. North Carolina macular dystrophy is unusual in its phenotypic variability and has been classified into different grades: grade 1, drusen-like yellow-white lesions in the macula at the level of the retinal pigment epithelium (RPE); grade 2, confluent drusen with or without pigmentary changes, RPE atrophy, or a disciform scar; and grade 3, staphyloma or coloboma with chorioretinal atrophy [13–15] (Figure 1). In grade 3 disease, hyperpigmentation and heaped-up white scar tissue are seen at the edge of the macular lesion. Development of choroidal neovascularization is uncommon but can worsen otherwise relatively stable visual acuity. Although grade 3 macular lesion is characteristic of NCMD, there has been much confusion in the literature regarding its nomenclature and 3-dimensional configuration. Small et al. [14] originally described these lesions as colobomas. Frank et al. [16] further studied this family and called these lesions choroidal atrophy, thereby creating some controversy. Gass [17] later described other members with NCMD as being “staphylomatous appearing.” Small et al. [13] reexamined the original family and concurred with Gass, because the cases were “clearly excavated, colobomatous, or staphylomatous in appearance when examined with binocular methods”. Subsequently, grade 3 lesions were labeled and characterized as colobomas or staphylomas in the literature [15]. However, terms such as *coloboma *and *staphyloma *clearly are misnomers; *coloboma *refers to an absence of tissue that had never been present, and *staphyloma *refers to an outpouching of the sclera that is lined with uveal tissue. On review of the published literature, there has never been proof of staphyloma formation by ultrasound. In the patients with staphylomatous changes, SD-OCT showed no outpouching of the scleral tissue. Instead, on biomicroscopic and binocular ophthalmoscopic examination, there is an illusion of outpouching because of the heaped-up subretinal gliosis and fibrosis surrounding the zone of atrophy in a curvilinear pattern. Khurana et al. [15] agree with Small's initial observation of a “posterior bowing of the central macula that produced a crater-like or staphylomatous appearance. The sharply demarcated temporal edge has a ledge or overhang affect”. Khurana proposed a new term for these grade 3 lesions, macular caldera. Caldera refers to a craterlike volcanic feature formed by the central collapse of land after a volcanic eruption. In our series, the SD-OCT scans of the 10 eyes with grade 3 lesions show the excavation centrally with the subretinal fibrous or gliotic tissue surrounding the atrophy, confirming that the term coined by Khurana might describe the best this lesion (Figure 1(c)). Although the pathogenesis of these grade 3 lesions is not known, Khurana proposed the following possible mechanism to describe the caldera formation. In NCMD, pathologic processes similar to those of AMD seem to begin in utero. In some cases, CNV, RPE tears, macular hemorrhaging, and eventually disciform-type lesions with fibrosis, gliosis, or both may occur. The characteristic greyish-white, heaped-up subretinal tissue that occurs at the border of the profound macular atrophy may be secondary to eruptive hemorrhaging and, subsequently, to accentuated fibrosis and gliosis and scar tissue formation. As the hemorrhage becomes organized, the authors speculate that the tremendous, inherent growth potential that characterizes virtually all fetal and neonatal tissues could result in the circumferential, heaped-up hyperplastic or hypertrophic subretinal scar.

*ABCA4* is the only gene known to cause autosomal recessive Stargardt disease. Localized to the rims of rod and cone outer segments, *ABCA4* gene product (an ATP-binding cassette transporter) normally transports all-trans-retinol produced in light exposed photoreceptor outer segments to the extracellular space [11, 18]. When a mutation in the *ABCA4* gene results in a dysfunctional protein, A2E (N-retinylidene-N-retinyl-ethanolamine), derived from the retinoid component of photopigment accumulating from photoreceptor OS debris, accumulates within the photoreceptors outer segment cell membrane. Adjacent RPE cells then phagocytose shed photoreceptor outer segments engorged with cytotoxic A2E, and large amount of intracellular A2E quickly accumulates, with flecks formation. The accumulation of flecks in Stargardt patients in our series progressed spatially from the fovea in a radial pattern, suggesting that the earliest events leading to fleck formation occur close to the fovea [19]. Fleck lesions on FAF imaging were observed to increase in hyperautofluorescence, reach a peak, then decrease subsequently in autofluorescence to near-background levels, and eventually become hypoautofluorescent. The hyperreflective deposits at the level of the ONL (type 2) could be the residual cover of the dome-shaped lesions (type 1). This latter hypothesis is consistent with the natural history of the flecks, which progressively degrade, from a well-defined lesion to residual material.

A2E laden RPE exhibits impaired degradation of phospholipids, which induces the release of proapoptotic proteins from mitochondria [18] and deterioration with the breakdown of cellular membranes and the ultimate lysis and death of RPE cell which may culminate in atrophy. ICGA may provide useful information about alterations of the choroid and the choriocapillaris [11]. The finding of hypocyanescence by ICGA in advanced disease (“dark atrophy” as previously described by Giani et al.) may be due to extended damage to choriocapillaris [20]. Moreover, the absence of normal choroidal hyperfluorescence by FA in the final stages, described by Yzer et al. [21] as “choroidal silence,” is due to marked atrophy of the choriocapillaris, which is a direct consequence of RPE absence. The choroid under the areas of atrophy seems to be morphologically intact.

Dark choroid is characterized by the absence of normal background fluorescence mainly due to the presence of RPE lipofuscin that absorbs the blue excitatory light [20]. 

Choroideremia is caused by mutations in the CHM gene localized to the long arm of X-chromosome (Xq21.2). The mutation affects the production of Rab escort protein isoform 1 (REP-1), the enzyme that plays a key role in the activation of Rab proteins that are responsible for the regulation of exocytic and endocytic cellular pathways. Boys have difficulty seeing at night in the first decade of life and then become aware of loss of peripheral vision by their teens. Areas of RPE disruption throughout the fundus are early clinical manifestations in a male patient with choroideremia (Figures 3(a), 3(b), and 3(c)). The progressive retinal changes in choroideremia can be described by careful analysis of SD-OCT images [22]. In the early phase, retinal thickening occurs with normal laminae, possibly due to Müller cell activation and hypertrophy creating interlaminar bridges. Late phases show shortening of the inner and outer segments, reduced thickness of the outer nuclear layer, and depigmentation of the RPE. Areas of chorioretinal atrophy become evident over time with loss of choriocapillaris, exposure of choroidal vessels, and loss of the RPE beyond the macula. In most cases central visual acuity is well maintained until quite late in the course of the disease, despite substantial loss of peripheral vision and significant chorioretinal degeneration [22].

Female choroideremia carriers can be identified clinically by the presence of patchy areas of RPE atrophy (Figures 3(d), 3(e), 3(f), and 3(g)) [23]. Fluorescein angiography has been advocated as a supplemental examination to detect female carriers by demonstrating window defect in the RPE. Furthermore, instead of an initial appearance of a choroidal flush, the choroidal circulation is delayed such that the retinal circulation and the choroidal circulation appear to fill simultaneously [24]. Fundus autofluorescence provides predictive information for the detection of female carriers. A characteristic pattern of granular high-density FAF with low-density fluorescence spots was present throughout the examined area in the female carriers included in this study. This pattern is due to the fact that the CMH gene product (REP1) is localized in rods and RPE cells, but not in cones [24]. Therefore, a patch of degeneration in a CMH carrier fundus may either arise from a clone of rod cells or RPE cells with the inactivated wild-type REP1 gene; X-linked disorders show independent degeneration of RPE cells and rods show irregularly distributed changes of hypo- (degenerating RPE) and hyperautofluorescence (degenerating rods).

Best vitelliform macular dystrophy was the first inherited retinal condition in which mutations in the *BEST1* gene were shown to be the underlying cause. Other mutations in the same gene are known to cause ARB. Hence this group of conditions is called *bestrophinopathies*. Common to all, the primary pathogenetic mechanism seems to be situated at the level of the RPE, with secondary photoreceptors involvement.

BVMD is caused by missense mutation in the *BEST1* gene, located on chromosome 11q13 and containing 11 exons. BEST1 encodes bestrophin-1, a protein localized to the basolateral surface of the RPE, where it forms chloride channels [25]. Even if fundoscopic appearance and progression of BVMD have been fully described, and FA and ICGA do not add any further significant information, the advent of novel imaging techniques such as fundus autofluorescence has confirmed that the yellowish intra- and subretinal material is hyperautofluorescent, due to its high lipofuscin content [26] (Figure 4). Data obtained with SD-OCT have suggested that material seen on fundoscopy as vitelliform material is located under the neurosensory retina rather than in and under the RPE [27]. Tomographic scans firstly show a thicker layer between the photoreceptors inner/outer segment interface and the underlying RPE (previtelliform stage). Material is subsequently deposited under the neurosensory retina (vitelliform stage) and is sometimes accompanied with a neurosensory detachment (pseudohypopyon stage), with abnormalities of the RPE and subsequent thinning of all retinal layers (vitelliruptive and atrophic stages). On the basis of OCT and FAF findings, Spaide et al. [27] has hypothesized a pathogenetic mechanism: inadequate removal of subretinal fluid leads to the physical separation of the photoreceptors from the underlying RPE; this results in the progressive accumulation of lipofuscin at the outer side of the neurosensory retina due to shedding of the outer segment discs without proper phagocytosis by the RPE cells; with time, atrophy of the photoreceptors and thinning of the accumulated material lead to a decrease of the hyperautofluorescence, eventually damage to the RPE ensues.

ARB is due to compound heterozygous or homozygous mutations in BEST1 and segregates as an autosomal recessive trait [28]. ARB has been hypothesized to represent the BEST1 null phenotype, with either very little or no functional bestrophin-1 available to RPE cells. Patients with ARB present with slowly progressive central visual loss, hypermetropia, irregularity of the RPE, and deep, scattered, white subretinal deposits that characteristically hyperfluoresce on FAF imaging [29] (Figure 5). Spectral-domain OCT shows RPE deposits, photoreceptor detachment, elongated and thickened photoreceptor OS, and preserved inner retinal layers.

Multimodal imaging of bestrophinopaties suggests a regional difference in expression of the bestrophin protein throughout the retina, with more protein being produced in the retinal periphery compared to the macula. This differential expression may lead to a more pronounced relative lack of functional bestrophin in the macula, which would explain why the latter is predominantly affected in BVMD.

Enhanced s-cone syndrome is a rare, slowly progressive autosomal recessive retinal degeneration related to mutation in NR2E3. NR2E3 encodes a ligand-dependent transcription factor that controls retinal progenitor cell fate. It promotes differentiation and survival of rod photoreceptors by differentially regulating transcription of rod- and cone-specific genes either directly or indirectly through interaction with other transcription factors. Mutation in NR2E3 is thought to cause disordered photoreceptor cell differentiation, possibly by encouraging default from the rod photoreceptor pathway to the s-cone pathway, thereby altering the relative ratio of cone subtypes [30]. Histopathologic data have reported an absence of rods, but a two-fold increase in the cone population, most of which were thought to be s-cones [31]. The retina was highly degenerated and disorganized. Photoreceptors were found only in the central and far peripheral regions. Densely packed cones were intermixed with inner retinal neurons [32]. The increased number of cones in ESCS is unique, since, as a group of disorders, the progressive retinal dystrophies are usually characterized by a loss of photoreceptors. The most typical ophthalmoscopic feature in our series was the presence of nummular, midperipheral pigmentary deposits at the level of the RPE (Figure 6). This typical retinal appearance was associated with an absence of FAF outside the vascular arcades, possibly relating to loss of photoreceptors in this region. A ring of relatively increased FAF in the transitional area between the region of absent FAF and the central zone of relatively normal FAF was evident. The increased FAF detected may be related to lipofuscin accumulation secondary to RPE-photoreceptor dysfunction in that area. Foveal schisis documented by FA and OCT was present in 6/7 patients, in keeping with previous findings. The typical nummular, midperipheral pigmentary changes are a useful clinical sign, but are not a consistent finding, being evident in 6/7 patients in the present series. The association of these nummular pigmentary deposits with white-yellow dots at the level of the RPE along the vascular arcades, focal hyperpigmentation within the arcades, and foveal or peripheral schisis is more suggestive of ESCS.

Fundus albipunctatus is considered to be a form of congenital stationary night blindness because of the predominant stationary nature of the disease and associated night blindness. It is caused by mutations in RDH5, encoding 11-cis-retinol dehydrogenase [33], and leads to impaired production of 11-cis retinal, restricting the photopigment turnover and leading to reduced formation of lipofuscin [34]. Therefore, the retinas of 2 patients in our series have a grainier appearance in FAF than normal retinas do [34, 35]. Recent studies support that the RPE has an alternative pathway involving RDH10 for the production of 11-cis-retinal, which can account for the delayed dark adaptation in patients with fundus and may explain the residual remaining FAF found in the youngest of our patient [35].

The presumed accumulation of cis-retinol and cis-retinyl esters in the RPE because of 11-cis-retinol dehydrogenase deficiency is responsible for the formation of white flecks in RDH5 mutation-associated fundus albipunctatus [36, 37] (Figure 7). It is not clear, however, why such accumulation should result in highly focal rather than diffuse accumulation of material. Furthermore, SD-OCT at high spatial resolution showed that the characteristic white flecks of the fundus are located in the outer retina at a specific depth, namely, in the photoreceptor inner and OS and RPE layers where the lesions are interspersed between normal-appearing stretches of outer retina, apparently bulging the outer limiting membrane [37].

In this study, we employed a digital retinal camera and a SD-OCT system combined with a multimodal cSLO topographic imaging system to obtain color fundus photographs and simultaneous recordings of cross-sectional SD OCT and c-SLO images, to correlate FA, FAF, and ICGA images. 

It should however be kept in mind that specialized retinal function and electrophysiologic testing are still fundamental to diagnose retinal genetic disease and to better understand the roles of various genes in maintaining structure and the function of the retina. For example, a normal fundus appearance and imaging do not preclude someone from carrying the disease *BEST1* gene, and an electrooculogram (EOG) should always be performed to look for a Light_peak_/Dark_through_ ratio generally less than 1.5 or 150%. As for ESCS, it must be noted that ERG recordings firstly showed that the large a-waves were nearly entirely driven by the s-cones, thus suggesting for the first time that the hypersensitivity and hyperfunction of the s-cone system were due to an increased number of s-cones.

In conclusion, genetic diseases may target the rod system or the cone system, together or independently, and multimodal imaging and electrophysiologic testing may be employed to determine the relative involvement of these two systems. As various therapies, including gene replacement, are proposed for these disorders, retinal function and imaging testing should help determine whether these treatments are beneficial in altering the natural course of a disease and whether they adversely affect retinal function.
